# RNO3 QTL regulates vascular structure and arterial stiffness in the spontaneously hypertensive rat

**DOI:** 10.1152/physiolgenomics.00038.2021

**Published:** 2021-11-10

**Authors:** Eric E. Morgan, Michael P. Morran, Nicholas G. Horen, David A. Weaver, Andrea L. Nestor-Kalinoski

**Affiliations:** ^1^Department of Surgery, College of Medicine and Life Sciences, University of Toledo, Toledo, Ohio; ^2^Department of Medicine, College of Medicine and Life Sciences, University of Toledo, Toledo, Ohio; ^3^University of Toledo Advanced Microscopy & Imaging Center, College of Medicine and Life Sciences, University of Toledo, Toledo, Ohio; ^4^Department of Radiology, Nationwide Children’s Hospital, Columbus, Ohio

**Keywords:** animal model, arterial stiffness, hypertension, SHR, vascular biology

## Abstract

Increased arterial stiffness is an independent risk factor for hypertension, stroke, and cardiovascular morbidity. Thus, understanding the factors contributing to vascular stiffness is of critical importance. Here, we used a rat model containing a known quantitative trait locus (QTL) on chromosome 3 (RNO3) for vasoreactivity to assess potential genetic elements contributing to blood pressure, arterial stiffness, and their downstream effects on cardiac structure and function. Although no differences were found in blood pressure at any time point between parental spontaneously hypertensive rats (SHRs) and congenic SHR.BN3 rats, the SHRs showed a significant increase in arterial stiffness measured by pulse wave velocity. The degree of arterial stiffness increased with age in the SHRs and was associated with compensatory cardiac changes at 16 wk of age, and decompensatory changes at 32 wk, with no change in cardiac structure or function in the SHR.BN3 hearts at these time points. To evaluate the arterial wall structure, we used multiphoton microscopy to quantify cells and collagen content within the adventitia and media of SHR and SHR.BN3 arteries. No difference in cell numbers or proliferation rates was found, although phenotypic diversity was characterized in vascular smooth muscle cells. Herein, significant anatomical and physiological differences related to arterial structure and cardiovascular tone including collagen, pulse wave velocity (PWV), left ventricular (LV) geometry and function, and vascular smooth muscle cell (VSMC) contractile apparatus proteins were associated with the RNO3 QTL, thus providing a novel platform for studying arterial stiffness. Future studies delimiting the RNO3 QTL could aid in identifying genetic elements responsible for arterial structure and function.

## INTRODUCTION

Increased stiffness of the large arteries is an independent risk factor for hypertension, stroke, and overall cardiovascular morbidity (1, 2). Systolic blood pressure (SBP) and the measure of pulse pressure (3) have emerged as superior indicators for evaluating cardiovascular risk and are now the targets of therapies that “de-stiffen” the vasculature by combining angiotensin-converting enzyme inhibitors with diuretics, as was done in the pREterax in regression of Arterial Stiffness in a contrOlled double-bliNd (REASON) study (4, 5). Understanding the factors that contribute to vascular stiffness is of major importance, and, to date, both clinical and animal models have found that arterial stiffness precedes hypertension (6–8). It remains important to continue to investigate new animal models that may offer insight into the mechanisms that control vascular stiffness that lead to the development of cardiovascular disease (CVD).

Previously published studies of arterial stiffness in the spontaneously hypertensive rat (SHR) strain (conducted before the onset of hypertension) suggest that there is a genetic predisposition to hypertension due to the aberrant organization of the elastin network that alters the mechanical nature of the arterial wall (9). Studies from our laboratory have demonstrated that heritable genetic variations encode physiological differences in vascular reactivity within the arterial wall (10). Specifically, we found increased vasoconstriction in SHR vessels compared with Brown Norway (BN) vessels in response to serotonin and prostaglandin F2α, and when the rat chromosome 3 (RNO3) alleles from the BN rat are introgressed to form the SHR.BN3 congenic strain, there is a further increase in vasoconstriction when compared with that observed in the parental SHR vessels (11). Given that increased vasoconstriction may lead to increased blood pressure, we sought to determine if the congenic SHR.BN3 strain demonstrates increased blood pressure compared with the parental SHR strain, and to further compare and characterize the relative stiffness, arterial wall structural components and extracellular matrix (ECM) of the SHR.BN3 and SHR strains using ultrasound and multiphoton microscopy (MPM). In addition, given that hypertension may alter both the structure and the function of the heart, we assessed progressive changes in left ventricular (LV) systolic function and geometry in the SHR.BN3 and SHR strains via two-dimensional (2D) transthoracic echocardiography. Characterization of blood pressure, arterial wall structure, and vascular and cardiac function of the SHR.BN3 allows us to begin delimiting our RNO3 region and provides a new model for evaluating differences in arterial stiffness. In turn, this will allow identification of the genetic elements responsible for the declining arterial function and remodeling and their role in predicting cardiovascular risk and assessment.

## METHODS

### Animal Strains

Inbred spontaneously hypertensive rats (SHR/NHsd) and Brown Norway (BN/SsNHsd) rat strains (Harlan Sprague–Dawley, Indianapolis, IN) were used to establish colonies maintained at the University of Toledo, Division of Laboratory Animal Resources. Hereafter, these strains will be referred to as SHR and BN. Breeding programs were approved by the Institutional Animal Care and Use Committee and complied with the National Institutes of Health “Guide for the Care and Use of Laboratory Animals.” All rats were housed with appropriate temperature and humidity controls with 12-h light/dark cycles and maintained on standard rat chow (Ralston Purina, Diet 5001) with ad libitum access to water. The congenic SHR.BN3 strain was developed as previously described (11). Briefly, the vascular neointimal growth quantitative trait loci 1–3 (Vnigr1-3 QTL) is a 39.3-megabase region on the q-arm of RNO3 between *D3Rat159* and *D3Rat1* containing >800 genetic elements that was identified in a strain screen for neointimal hyperplasia (11, 12). The quantitative trait loci (QTL) was confirmed using the SHR.BN3 congenic strain that incorporates the QTL region from the BN donor strain introgressed onto the SHR genetic background (11). A cumulative list of all genetic elements contained within the Vnigr1-3 QTL area that were introgressed onto the SHR background can be located in the Rat Genome Database Web Site (http://rgd.mcw.edu/, 04-16-2021 search of RNO3 138374177 bp–177699992 bp, https://tinyurl.com/57dnhke5). All presented experiments used male animals.

### Tissue Processing

Common iliac arteries were harvested from male SHR and SHR.BN3 for confocal microscopy and histology. Rats were perfused as previously described with 10% neutral buffered formalin and arteries were processed and embedded in paraffin (12). SHR.BN3 (*n* = 12) and SHR (*n* = 17) arteries were sectioned at 5 μm and stained with DRAQ5 (Deep Red Anthraquinone 5, Invitrogen, Carlsbad, CA) for multiphoton microscopy (MPM) analysis. Hematoxylin and eosin (H&E) staining and trichrome staining were performed on 5-µm serial sections of the SHR.BN3 (*n* = 12) and SHR (*n* = 17) arteries. Sections were scanned at ×20 on the Olympus VS120 virtual slide scanner with the OlyVIA software (Olympus Americas, Center Valley, PA) to confirm arterial morphology and integrity (Fig. 3).

### Confocal Microscopy

Images were acquired using a Leica TCS SP5 laser scanning confocal microscope (Leica Microsystems, Bannockburn, IL) equipped with a ti-sapphire tunable multiphoton laser (Coherent, Santa Clara, CA). Second-harmonic generation (SHG) and autofluorescence were used to view collagen and elastin (13). SHG for collagen was excited at 860 nm and emission collected at 425–435 nm (13). Elastin autofluorescence was excited at 760 nm and emission collected at 436–511 nm (13). DRAQ5 was imaged using 633/681 nm. Images were acquired at 512 × 512 format with 1 µm z-step size using sequential scan acquisition to ensure no spectral overlap.

### Collagen Bundling and Cell Counts

Confocal images acquired at ×40 were analyzed using MetaMorph 7.7.0.0 Imaging Software (Molecular Devices, San Jose, CA). Three collagen attributes were analyzed: total collagen area, collagen bundle area, and collagen bundle area/total collagen area. Arteries were imaged at three locations along the circumference and averaged. Total collagen area was obtained using the integrated morphometry analysis. Each collagen collection with an area of at least 100 pixels, area determined previously (data not shown), was summed to determine the collagen bundle area. The collagen bundle area was divided by the total collagen area to determine the relative amount of collagen bundling per artery. Using MetaMorph software, nuclei stained with DRAQ5 were counted at three locations within the tunica media and adventitia, and the counts were averaged.

### Blood Pressure

Male SHR.BN3 (*n* = 8) and SHR (*n* = 9) rats were age matched, and systolic blood pressure (SBP) was measured using the tail-cuff method. Blood pressure (BP) was acquired on conscious, trained rats warmed to 28°C. BP was recorded blinded, starting at 8 wk in 2-wk increments until 32 wk, using a noninvasive CODA high-throughput system (Kent Scientific, Torrington, CT) once a day for 2 consecutive days. BP values for each day were averaged, and the final BP value was the average of the 2 days.

### Echocardiography and Vascular Ultrasound

LV systolic function and geometry and aortic elasticity were assessed in SHR.BN3 and SHR rats using an Acuson Sequoia C512 ultrasound machine equipped with a 15-MHz linear transducer (Siemens Medical Solutions, Malvern, PA) as previously described (14, 15). Briefly, rats were anesthetized (1–2% isoflurane by O_2_ inhalation) before shaving the neck, chest, and left thigh. Animals were placed on warming pads in the supine position with electrodes. Two-dimensional M-mode LV images were obtained from the transthoracic parasternal long-axis window. Doppler studies of aortic and transmitral flows were performed from parasternal and foreshortened apical windows. The carotid and femoral arteries were scanned, and pulse wave Doppler signals were recorded. The distance between the sternal notch and the femoral artery at the point of probe applanation was measured. All data were analyzed blinded with on-board software. Fractional shortening (FS), relative wall thickness (RWT), cardiac output (CO), and aortic pulse wave velocity (PWV) were calculated as previously described (15).

### Western Blotting

The abdominal aorta from below the renal arteries and the common iliac arteries were harvested from 16-wk age-matched male SHR and SHR.BN3 animals (*n* = 4). Tissue was frozen on dry ice and ground using a mortar and pestle. Powdered tissue was lysed in ice-cold Mammalian Protein Extraction Reagent (MPER) containing 1:100 of both Halt Phosphatase (Thermo Fisher 78420) and Protease Inhibitor Cocktails (Thermo Fisher 87786) for 15 min on ice and then centrifuged for 15 min at 4°C. Supernatants were aliquoted and stored frozen at −80°C. Protein was quantitated with the Pierce BCA Protein Assay Kit (Thermo Fisher 23227). Equivalent amounts of protein (12.5 µg) were boiled for 5 min in sample reducing buffer and placed on ice. Samples and dual color ladder (Bio-Rad Precision Plus 5 µL) were run on precast Bio-Rad 12% gels and separated at 100 V for 45–60 min. Proteins were transferred to PVDF membranes using the Bio-Rad Transblot-Turbo Transfer system for 7 min. Membranes were blocked in Tris-buffered saline-0.1% Tween 20 (TBST) with 5% nonfat dry milk for 60 min at room temperature and washed three times for 5 min in TBST. Primary antibodies in TBST were incubated overnight at 4°C. Due to potential signal detection overlap, protein detection was carried out sequentially. Initial protein detection for normalization was carried out using the primary antibody glyceraldehyde-3-phosphate dehydrogenase (GAPDH; Proteintech 10494-1-AP) incubated at a 1:8,000 dilution overnight at 4°C. The membrane was washed three times for 5 min in TBST and incubated with horseradish peroxidase (HRP)-conjugated donkey-anti-rabbit IgG secondary antibody (Jackson ImmunoResearch, 711-035-152) at 1:2,000 dilution for 60 min, followed by three additional 5-min wash steps before band visualization. Bio-Rad Clarity Western ECL Substrate for chemiluminescence detection was added and visualized using the Syngene G-Box system (Syngene, Frederick, MD). Upon completion of GAPDH detection, the membrane was washed three times for 5 min in TBST, before a 10-min 37°C incubation in Restore Western Blot Stripping Buffer (Thermo Fisher Cat. No. 21059). After stripping, the membrane was washed three times for 5 min in TBST and reblocked in 5% nonfat dry milk. Additional protein detection was carried out using the primary antibody actin α-2, smooth muscle (ACTA2; Proteintech 14395-1-AP) incubated at 1:1,000 dilution overnight at 4°C. Secondary antibody incubation and band detection were carried out as previously described for GAPDH. Images were analyzed in the Syngene GeneTools software to determine band densitometry values. Individual densitometry values were normalized against GAPDH values before averaging and statistical analysis.

### Immunohistochemistry

Paraffin-embedded arterial sections of the SHR.BN3 (*n* = 12) and SHR (*n* = 17) were cut at 5 µm, deparaffinized, and tissue hydrated. Tissues were permeabilized in TBST with 0.05% Tween-20 for 30 min. Tissues were then blocked in Tris-buffered saline (TBS) containing 10% horse serum for 2 h at room temperature and washed in TBS for 5 min. Antinuclear protein Ki67 (Ki67) antibody (Abcam, ab15580) was diluted 1:100 in TBS with 10% horse serum overnight at 4°C. Tissue was washed twice with TBST for 5 min. Slides were then incubated in 0.3% H_2_O_2_ in TBS for 15 min at room temperature, followed by a 5-min wash in TBS. Biotinylated anti-rabbit IgG antibody (Vector BA-1000) was diluted 1:200 in TBS and incubated on tissue for 1 h at room temperature. Secondary antibody was removed, and slides were washed in TBS for 5 min. ABC reagent (VECTASTAIN Elite PK-7100, Vector Laboratories, Burlingame, CA) was applied for 30 min at room temperature and washed for 5 min in TBS. Colorimetric development was carried out using the reconstituted SIGMAFAST diaminobenzidine (DAB) tablets (Sigma-Aldrich, D4293) for 1–3 min. DAB development was quenched in TBS and counterstained in hematoxylin, dehydrated, and mounted with Eukitt (Electron Microscopy Sciences) mounting media. Sections were scanned at ×20 on the Olympus VS120 virtual slide scanner with the OlyVIA software (Olympus Americas, Center Valley, PA).

### Isolation of Primary Vascular Smooth Muscle Cells

SHR and SHR.BN3 rats were euthanized and abdominal aorta and both the left and right common iliac arteries isolated. Tissue was placed in ice-cold phosphate buffered saline (PBS) to remove residual blood and washed three times. Tissue was opened longitudinally with fine-tip microdissecting scissors. The endothelium was scrapped gently with a scalpel and cut into small 1-mm^2^ pieces. Tissues were transferred to 15-mL conical tubes and washed three times in warmed complete media, followed by trypsinization (0.25% Trypsin-EDTA Gibco 25200-056) on a rocking platform two times for 5 min each. Fragments were washed twice in 37°C complete media to remove any residual trypsin. Complete media consisted of Media 231 (Gibco M-231-500) supplemented with 25 mL of 20× Smooth Muscle Growth Supplement (SMGS; Gibco S-007-25) and 5 mL of 100× antibiotic-antimycotic (Gibco 15240). Individual tissue pieces were transferred to adhere to a T-25 flask. Flasks remained upright in a 37°C incubator for 4–6 h to allow firm adherence to the flask. Flasks were then placed horizontally, and 4 mL of complete media was gently added. Flasks were left completely undisturbed for ∼3 days before initial visual inspection. Media was changed and refreshed as needed until vascular smooth muscle cell (VSMC) growth was seen. Confluent flasks were trypsinized and passaged before experimentation.

### Immunofluorescence Staining

VSMCs from three individual animals of the SHR.BN3 and SHR strains were grown on glass coverslips in a 24-well plate and allowed to reach 40%–70% confluence. Cells were washed in PBS before fixation with 4% paraformaldehyde in PBS for 15 min. Samples were washed for 2 min for a total of three washes with PBS. Cells were permeabilized with 0.25% Triton X-100/PBS (PBST) for 10 min at room temperature. Samples were washed for 2 min for a total of three washes before blocking with 1% BSA in PBST for 30 min. Samples were washed for 2 min for a total of three washes and the primary antibody incubated overnight (12–16 h) at 4°C in 1% BSA-PBST at 1:200 (ACTA2, Proteintech Cat. No. 14395-1-AP). Samples were washed for 2 min for a total of three washes before secondary antibody addition in 1% BSA-PBST. Secondary antibody (Alexa Fluor Plus 488, Thermo Fisher Cat. No. A32790) was diluted to 1:2,500 and incubated for 60 min at room temperature followed by three washes in PBS. Cells were counterstained with DAPI (4′,6-diamidino-2-phenylindole) and mounted with Fluoromount-G (SouthernBiotech, Birmingham, AL). Slides were imaged on the Leica TCS SP5 laser scanning confocal microscope (Leica Microsystems, Bannockburn, IL) as described earlier.

### Rat LncRNA Expression Microarrays

RNA microarray sample prep was carried out on vascular smooth muscle cells (VSMCs) of SHR and SHR.BN3 at *passage 2*. Approximately 1.1 × 10^6^ cells were lysed, and RNA was extracted using the RNeasy Mini Kit (Qiagen Cat No. 74104) as per the manufacturer’s instructions. Samples were aliquoted and frozen directly at −80°C. A quality control for sample quantity and purity was performed and quantitated via a nano drop. RNA integrity was assessed by performing denaturing agarose gel electrophoresis for gDNA contamination. Frozen individual samples of RNA (∼4 µg total) for each SHR and SHR.BN3 cell type were shipped overnight on dry ice to Arraystar Inc. (Rockville, MD) for RNA microarray services.

Microarray analysis was performed via Agilent Array. Sample preparation and microarray hybridization were performed based on standard manufacturer protocols with minor modifications (16). Samples were amplified and transcribed into fluorescent cRNA without a 3′-bias using a random priming method (Flash RNA Labeling Kit, Arraystar Inc.). Labeled cRNAs were hybridized to the Rat LncRNA Array v2.0 (4 × 44 K, Arraystar Inc.) and scanned by the Agilent Scanner G2505C. Acquired array images were analyzed via the Agilent Feature Extraction software (v. 11.0.1.1). Quantile normalization and subsequent data processing were performed using the GeneSpring GX v12.1 software package (Agilent Technologies). Differentially expressed LncRNAs and mRNAs were identified through fold change filtering. KEGG (Kyoto Encyclopedia of Genes and Genomes) pathway analysis was applied to determine the roles these differentially expressed mRNAs played in these biological pathways. Finally, hierarchical clustering was performed to show the distinguishable LncRNAs and mRNAs expression pattern among samples.

### Statistical Analysis

Statistical analyses were performed using one-way ANOVA, with results presented as means ± SD. Differences between strains were determined using Student’s *t* test, with results presented as bar graphs or scatter dot plots with lines corresponding to means ± SE. A *P* < 0.05 was considered to be significant. The agreements between inter- and intraobserver PWV measurements were tested by Bland–Altman analysis. All analyses were performed with GraphPad Prism software (GraphPad Software, Inc., San Diego, CA).

## RESULTS

### Blood Pressure and Arterial Elasticity

**Figure 1.**
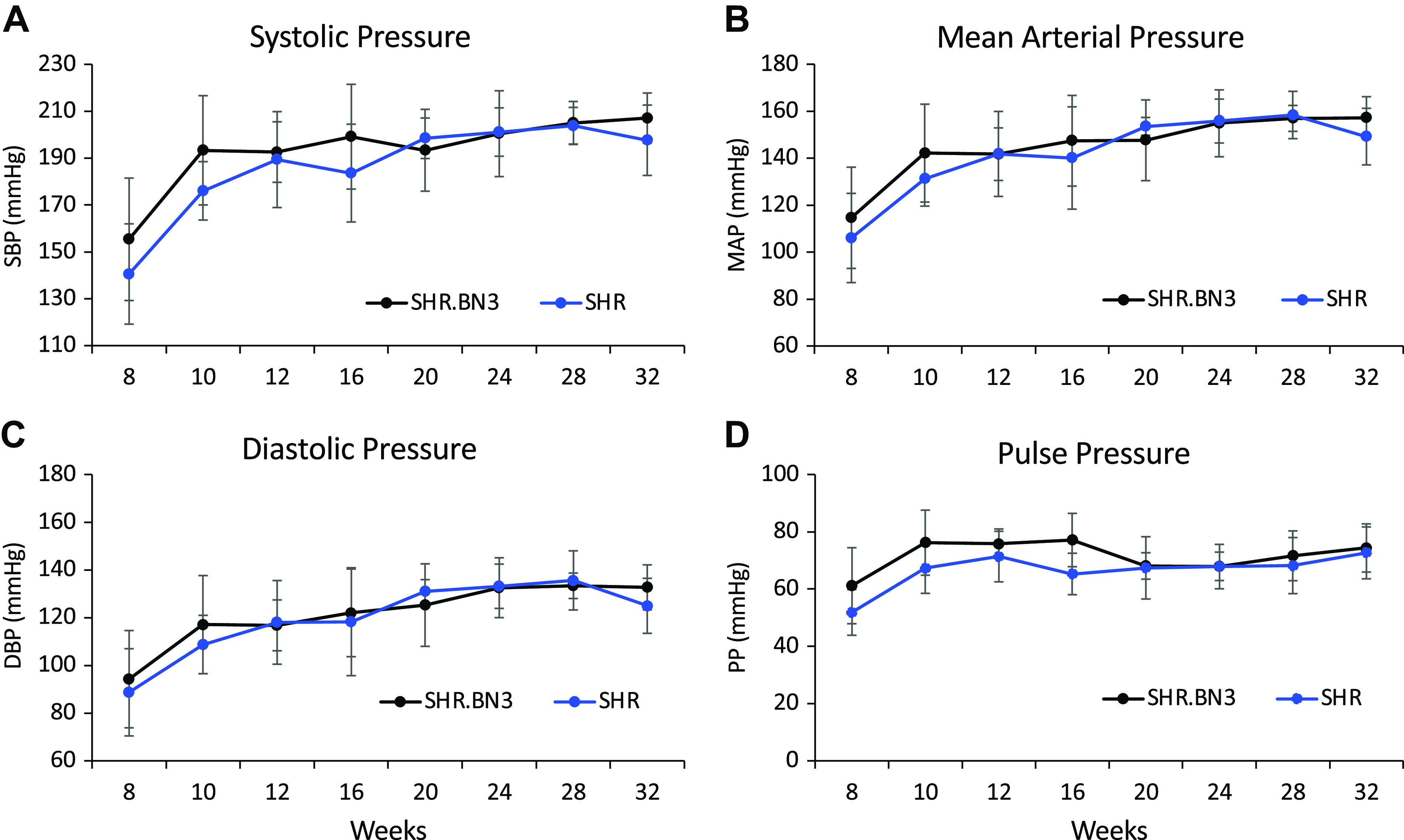
BP in SHR (*n* = 9) and SHR.BN3 (*n* = 8) strains. *A*: systolic blood pressure. *B*: mean arterial pressure. *C*: diastolic blood pressure. *D*: pulse pressure. BP data are means ± SD (*P* < 0.05, statistically significant). Pulse pressure = SBP − DBP and mean arterial pressure = DBP + [⅓(SBP − DBP)]. BP, blood pressure; DBP, diastolic blood pressure; MAP, mean arterial pressure; PP, pulse pressure; SBP, systolic blood pressure; SHR, spontaneously hypertensive rat.

Previous comparison of the SHR and SHR.BN3 strains identified significant differences in vasoreactivity (11), and we sought to determine if these differences were accompanied by differences in BP. We, therefore, measured BP via tail cuff in the SHR (*n* = 9) and SHR.BN3 (*n* = 8) animals starting at 8 wk of age, repeating the measurements every 2 wk until 32 wk of age (Fig. 1). To our surprise, no differences were seen in systolic, diastolic, mean arterial pressure, or pulse pressure between the strains at any time point from 8 to 32 wk of age (Fig. 1, *A–D*).

**Figure 2.**
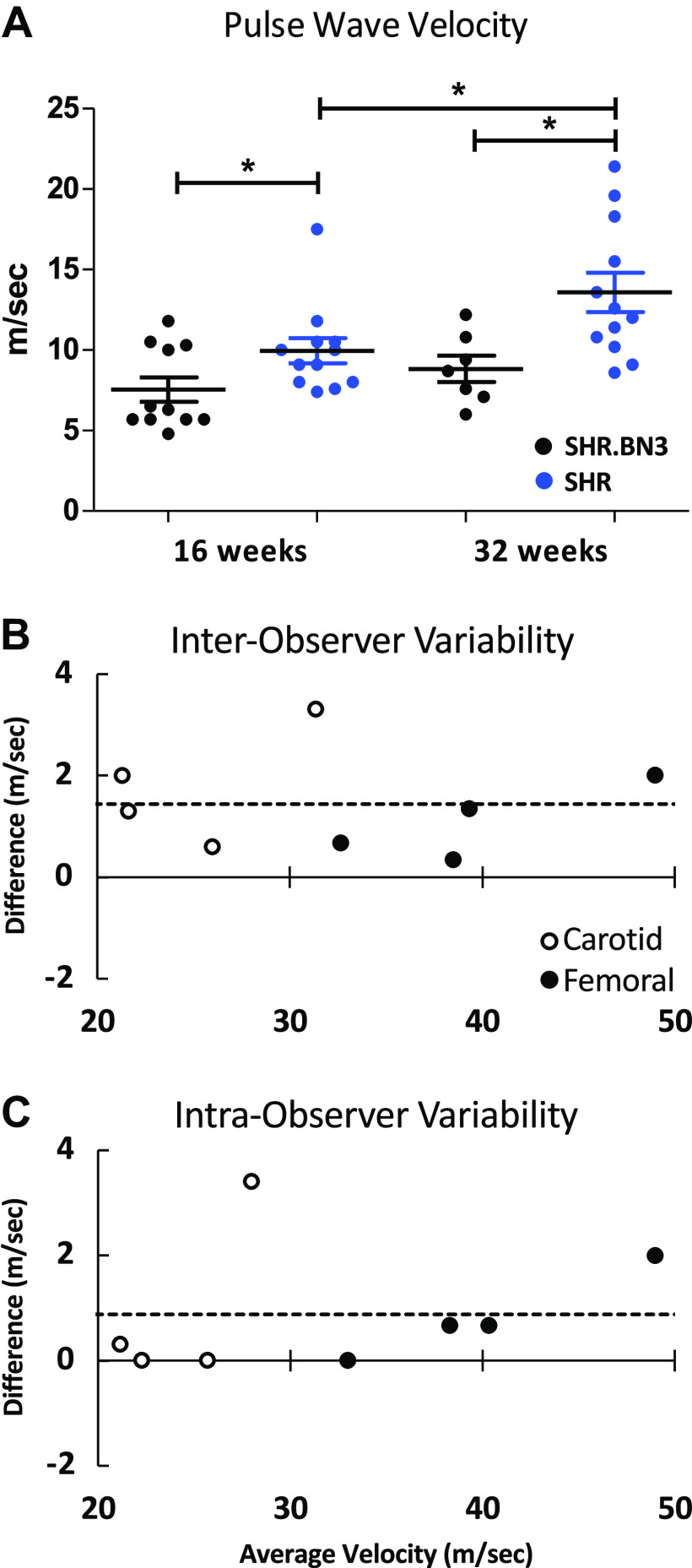
Pulse wave velocity (PWV) measure of arterial stiffness. *A*: PWV in SHR.BN3 (black) and SHR (blue) strains at 16 (*n* = 11, 12) and 32 wk (*n* = 7, 12). Data are means ± SE (*P < 0.05, statistically significant). Interobserver (*B*) and intraobserver (*C*) agreement measures of velocity. Open circles and solid circles corresponding to carotid and femoral velocity measure differences, respectively. SHR, spontaneously hypertensive rat.

We also evaluated arterial stiffness using PWV as previously described (14). PWV was measured at 16 and 32 wk, with no difference in BP or heart rate at either time point. The average PWV at 16 wk was significantly faster in the SHR strain at 9.8 m/s (*n* = 12) compared with 7.6 m/s (*n* = 11) in the SHR.BN3 strain (Fig. 2*A*, *P* < 0.05). A significant difference in PWV was also found at 32 wk between the SHR (12.79 m/s, *n* = 12) and the SHR.BN3 (8.92 m/s, *n* = 7; Fig. 2*A*, *P* < 0.05). Interestingly, the SHR strain also showed a significant increase in PWV from 16 to 32 wk (Fig. 2*A*, *P* < 0.05), whereas the SHR.BN3 strain did not. To assess intra- and interobserver variability of our wave propagation time measures, PWV was recalculated in a randomly selected subset of animals (*n* = 4 per group) by two independent observers under the same conditions (14). One observer analyzed the data twice on separate days to determine intraobserver variances. Intra- and interobserver variability were calculated as the absolute value of the differences between the two observations divided by the average of the two observations. Figure 2, *B* and *C*, shows the Bland−Altman plot for the percentage of the mean difference and 95% limits for inter- and intraobserver measurements of the carotid (open circles) and femoral (solid circles) time measurements, demonstrating a statistically significant relationship between readings (*P* < 0.0001) with very good agreement.

### Vascular Wall Parameters

**Figure 3.**
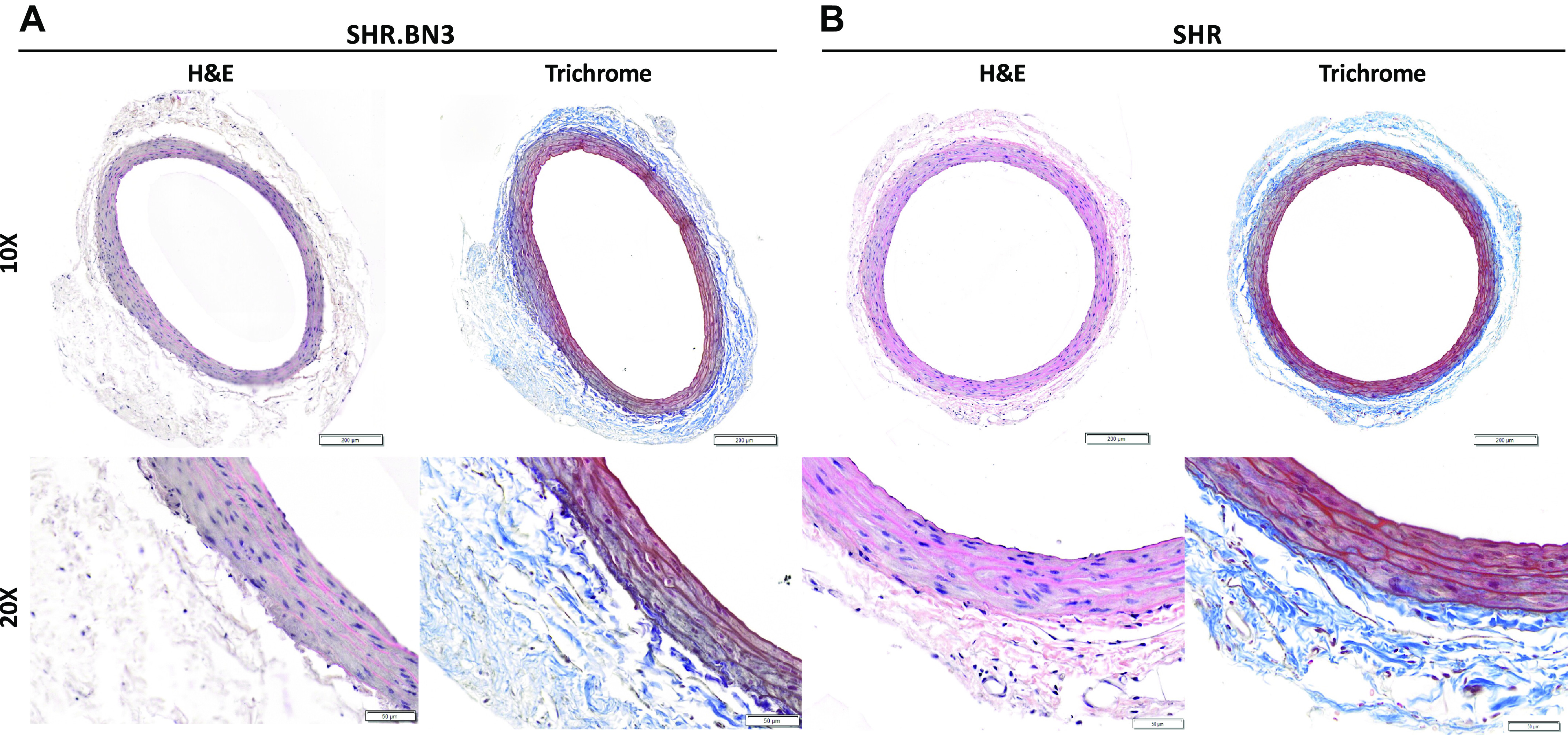
H&E and trichrome staining of formalin-fixed paraffin-embedded arterial cross sections to determine histological morphology of the SHR.BN3 (*A*) and SHR (*B*) strains. Brightfield images were acquired on an Olympus VS120 slide scanner at ×10 and ×20 magnification. Scale bars are 200 μm in the ×10 magnification and 50 μm at ×20 magnification. H&E, hematoxylin and eosin; SHR, spontaneously hypertensive rat.

**Figure 4.**
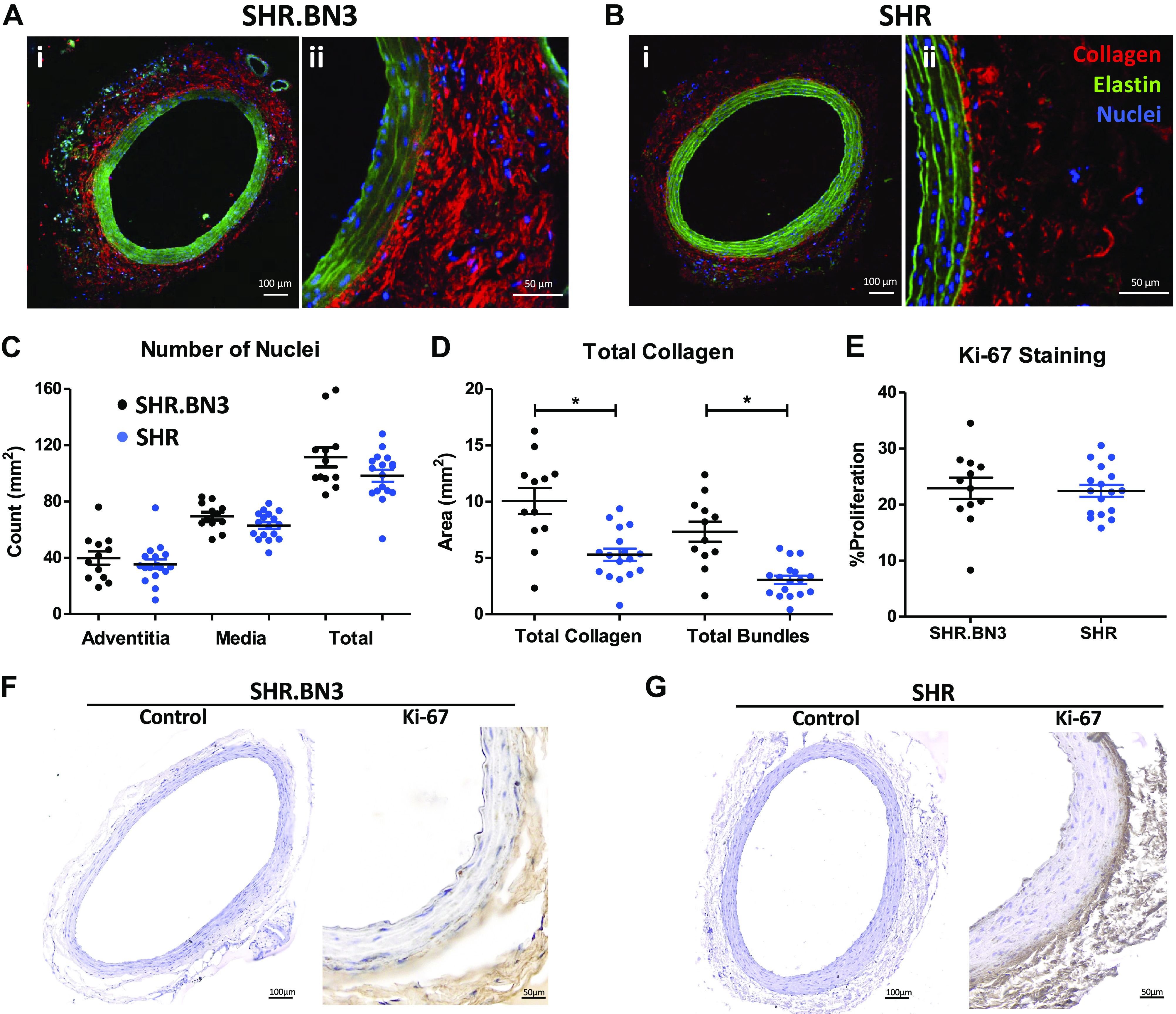
MPM imaging of arterial sections. SHR.BN3 (*A*) and SHR (*B*) arterial cross section at ×10 (*i*) and ×40 (*ii*) magnification. Nuclei are labeled with DRAQ5 (blue), elastin (green), and collagen (red). *C*: average number of nuclei in the adventitia and media of arteries in the SHR.BN3 (*n* = 12) and SHR (*n* = 17). *D*: quantification of collagen and collagen bundles in arteries. *E*: immunohistochemical detection and quantification of Ki-67 in formalin-fixed paraffin-embedded arterial cross sections of SHR.BN3 (*F*; *n* = 12) and SHR (*G*; *n* = 17). Slides were stained with DAB and hematoxylin (positive cells brown, nucleus blue). Scale bars equal 100 µm and 50 µm as indicated. Values are expressed as means ± SE. *P < 0.05, statistically significant. DAB, diaminobenzidine; DRAQ5, Deep Red Anthraquinone 5; Ki-67, nuclear protein Ki-67; MPM, multiphoton microscopy; SHR, spontaneously hypertensive rat.

We visualized arterial wall structural properties using H&E and trichrome staining of formalin-fixed paraffin-embedded arterial cross sections (Fig. 3) and MPM (Fig. 4). The intima, media, and adventitia were defined by the internal and external elastic lamina borders (Fig. 3 and Fig. 4, *A* and *B*, green). The adventitia was defined by the area outside the external elastic lamina with predominately collagen labeling (Fig. 4, *A* and *B*, red). The media extends from the internal to external elastic lamina borders (Fig. 4, *Aii* and *Bii*, green) with nuclei labeled with DRAQ5 (blue). Nuclei in the media and adventitia were counted in arteries of SHR.BN3 (*n* = 12) and SHR (*n* = 17), with no differences in either the media or adventitia (Fig. 4*C*). Collagen was quantified as total area and as bundled fibrils per square micrometer (Fig. 4*D*), with a significant increase in total collagen and bundled fibrils in SHR.BN3 compared with SHR rats. To determine the proliferation index for the VSMCs in both strains, we stained arterial cross sections of the same arteries for the proliferation marker Ki-67 (Fig. 4, *E–G*). There was no difference in proliferation between strains with an index of 22% with staining predominately in the nuclear region (Fig. 4, *F* and *G*, brown).

### Left Ventricular Function and Hypertrophy

**Figure 5.**
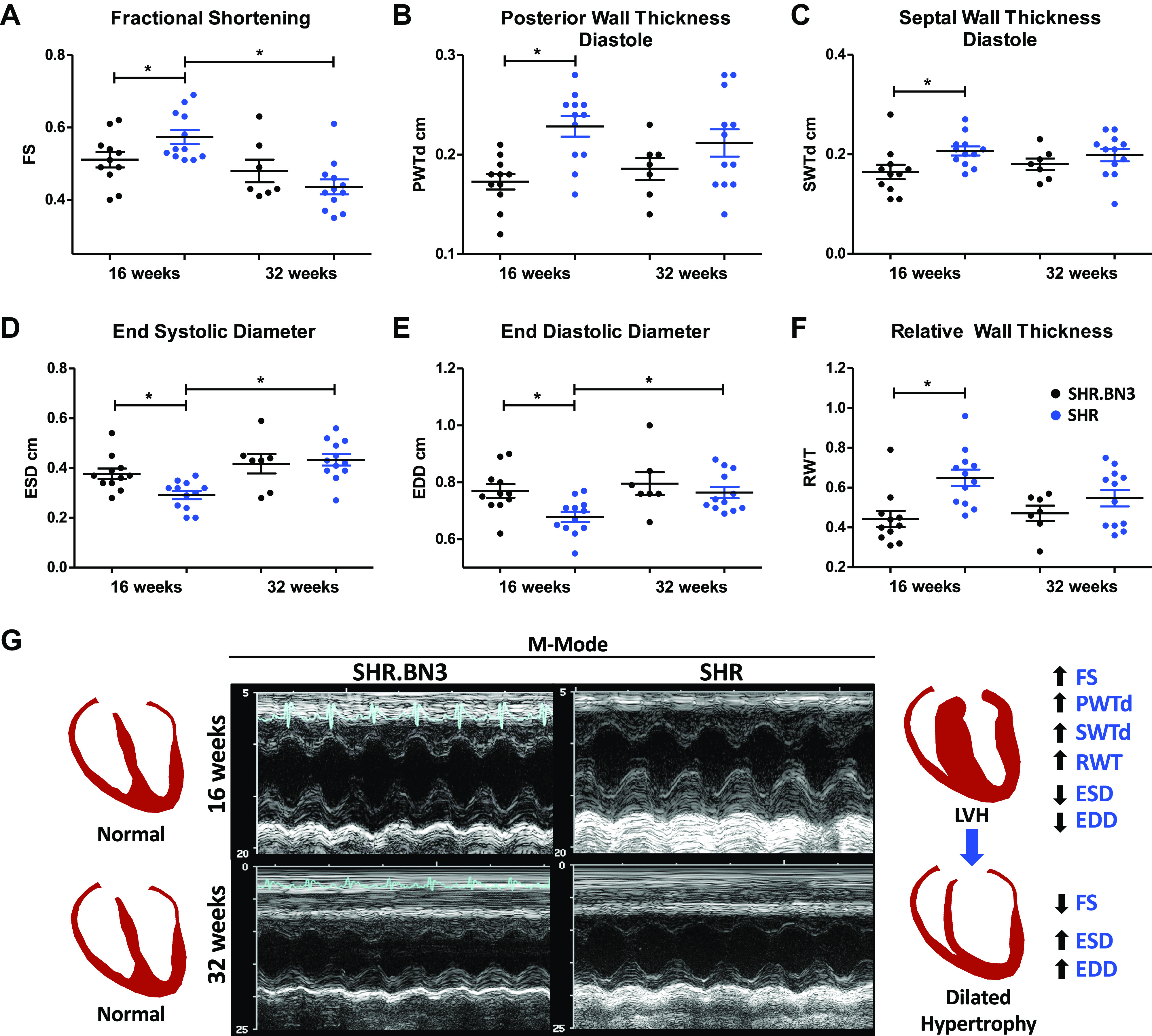
Echocardiography analysis in SHR.BN3 (black) and SHR (blue) at 16 (*n* = 11, 12) and 32 (*n* = 7, 12) wk. *A*: fractional shortening. *B*: posterior wall thickness diastole. *C*: septal wall thickness diastole. *D*: end systolic diameter. *E*: end diastolic diameter. *F*: relative wall thickness. Values are expressed as means ± SE. *P < 0.05, statistically significant. *G*: M-mode at 16 and 32 wk demonstrating normal LV geometry and function in the SHR.BN3 compared with cardiac hypertrophy (16 wk) progressing to dilation (32 wk) in the SHR. EDD, end diastolic diameter; ESD, end systolic diameter; FS, fractional shortening; LV, left ventricular; LVH, left ventricular hypertrophy; PWTd, posterior wall thickness diastole; RWT, relative wall thickness; SHR, spontaneously hypertensive rat; SWTd, septal wall thickness diastole.

**Figure 6.**
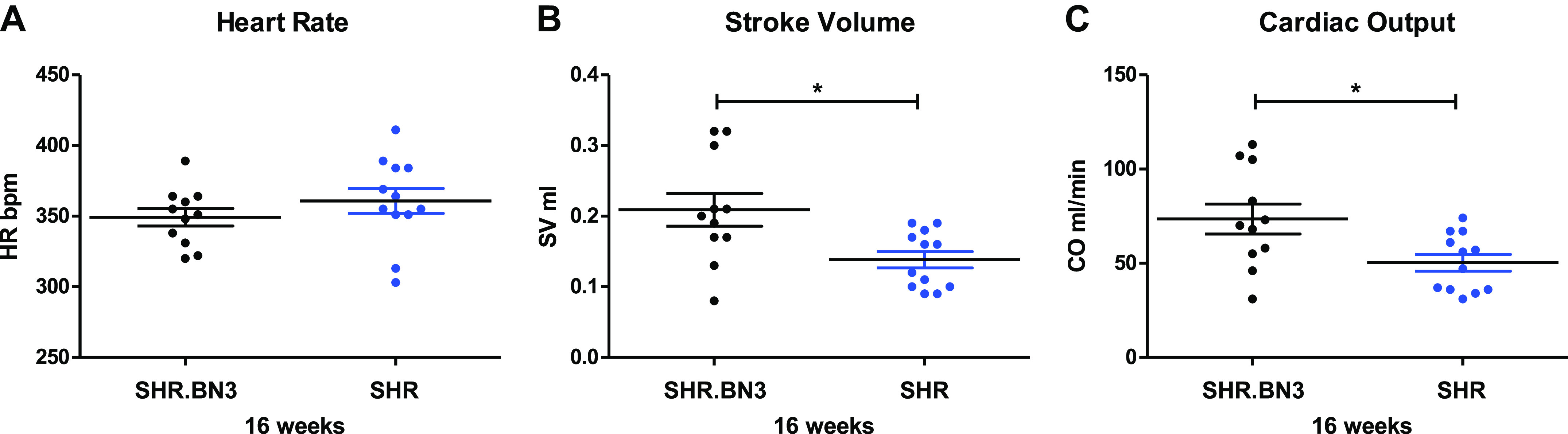
Echocardiography analysis in SHR.BN3 (black, *n* = 11) and SHR (blue, *n* = 12) at 16 wk. *A*: heart rate. *B*: stroke volume. *C*: cardiac output. Values are expressed as means ± SE. *P < 0.05, statistically significant. CO, cardiac output; HR, heart rate; SHR, spontaneously hypertensive rat; SV, stroke volume.

Arterial stiffness is associated with left ventricular hypertrophy (LVH) and dysfunction in rat models of hypertension (17). In the present study, we found that at 16 wk, SHR rats demonstrated increased systolic function, as evidenced by greater fractional shortening (FS; Fig. 5, *A* and *G*, *P* < 0.05). LV posterior wall thickness (PWT) and septal wall thickness (SWT) were both significantly increased and LV diameter at end systole (LVESD) and LV diameter at end diastole (LVEDD) were significantly decreased in the SHR rats, leading to a significantly increased RWT at the 16-wk time point (Fig. 5, *B–G*, *P* < 0.05). At 32 wk of age, however, there was no difference in FS between SHR and SHR.BN3 rats, although FS was significantly reduced in the SHR animals at 32 wk of age when compared with 16 wk of age (Fig. 5, *A* and *G*, *P* < 0.05). Similarly, there were no differences in PWT, SWT, LVESD, LVEDD, or RWT between the 32-wk-old strains, although both LVESD and LVEDD were significantly increased in the SHR rats at 32 wk compared with 16 wk, with a concomitant significant decrease in RWT (Fig. 5, *D–G*, *P* < 0.05). Doppler-derived echocardiographic assessment of cardiac function in 16-wk-old animals demonstrated increased systolic function, as evidenced by significantly higher stroke volume and CO in the SHR.BN3 with no difference in heart rate (Fig. 6, *A–C*, *P* < 0.05).

### Vascular Smooth Muscle Cell Phenotypic Diversity

**Figure 7.**
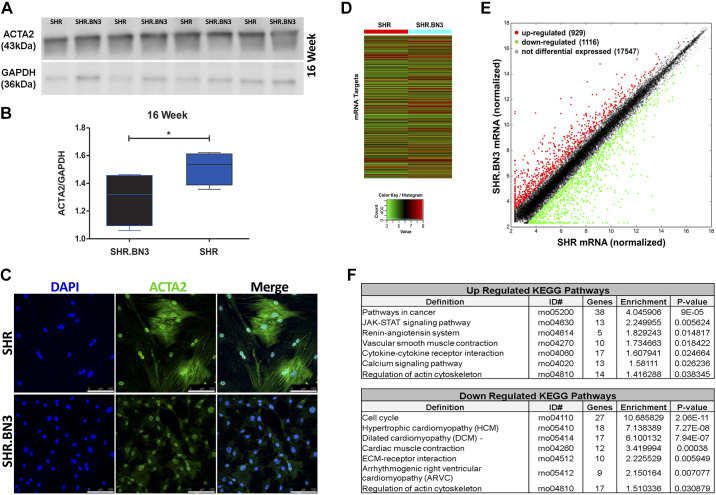
VSMC phenotypic diversity. *A*: Western blot comparison of ACTA2 and GAPDH in arterial tissue. *B*: box and whisker plot of normalized ACTA2 in SHR.BN3 (blue) and SHR (black) arterial tissue (*P < 0.05, statistically significant). *C*: immunofluorescence staining of ACTA2 in primary VSMCs in vitro from SHR.BN3 and SHR*. D*: hierarchical clustered heat map of differentially expressed mRNA targets from VSMCs. Color scale values for high relative expression (red) and low relative expression (green). *E*: scatter plot of normalized mRNA targets. Red indicates upregulated gene targets, green indicates downregulated gene targets, and gray indicates nondifferentially expressed targets. The gray dashed line indicates the threshold of a ±2.0 fold change. *F*: KEGG pathway analysis of differentially expressed mRNA targets of the SHR.BN3 to SHR comparison. *P* < 0.05 was determined using Fisher’s exact test with the enrichment score of each pathway ID equal to “−log10 (*P* value).” The genes column indicates the total number of differentially expressed genes in the given pathway ID. ACTA2, actin α-2, smooth muscle; ARVC, arrhythmogenic right ventricular cardiomyopathy; DCM, dilated cardiomyopathy; GAPDH, glyceraldehyde-3-phosphate dehydrogenase; HCM, hypertrophic cardiomyopathy; SHR, spontaneously hypertensive rat; VSMC, vascular smooth muscle cell.

Major contributors to arterial stiffness are the ECM and VSMCs controlling mechanical load, contraction, and mechanotransduction. Therefore, we measured the actinomyosin cytoskeletal protein ACTA2 (actin α-2, smooth muscle) that controls VSMC phenotypic diversity that results in arterial stiffness and strength (18–20). Protein from arterial tissue of individual SHR and SHR.BN3 rats at 16 wk (*n* = 4) was compared by Western blot. The SHR strain had a significant increase in ACTA2 compared with the SHR.BN3 strain (Fig. 7, *A* and *B**, P* < 0.05). Furthermore, we cultured VSMCs isolated from 16-wk-old SHR and SHR.BN3 rats and stained for ACTA2 in the actin cytoskeletal network. The ACTA2 staining in the SHR showed a dramatic and distinct actin cytoskeletal framework compared with SHR.BN3-derived cells (Fig. 7*C*), corresponding to a VSMC contractile phenotype. In addition, VSMCs were grown to confluence and harvested for RNA extraction at *passage 2* to compare the global gene expression profile of both LncRNAs and known coding mRNAs on the same array. The Arraystar Rat Microarray was carried out to identify differences in LncRNAs (*n* = 13,611) and protein-coding transcript mRNAs (*n* = 24,626). A hierarchical clustered mRNA heat map indicates the expression differences between the isolated VSMCs for each strain. Green color indicates low mRNA expression, whereas red indicates high gene expression values (Fig. 7*D*). Differential expression analysis of the SHR.BN3 to SHR VSMCs (Fig. 7*E*) shows 929 upregulated mRNAs (red) and 1,116 mRNAs downregulated mRNAs (green). Differentially expressed mRNAs were imported into the KEGG pathway database. Select KEGG pathways are presented based on *P* value and enrichment score and provide a framework as to the mechanistic regulation of the VSMC associated with the RNO3 QTL region (Fig. 7*F*). Interestingly, the KEGG analysis identifies networks associated with vascular structure and stiffness-associated pathology (20, 21), including collagen deposition, renin-angiotensin signaling, actin cytoskeleton regulation, cell cycle, and ECM interactions.

## DISCUSSION

Increased stiffness of the large arteries is an independent risk factor for hypertension, stroke, and overall cardiovascular morbidity (1). Thus, understanding the factors that contribute to vascular stiffness is of major importance. PWV is a measure of arterial stiffness (22), and elevated PWV is an independent marker of cardiovascular risk in human hypertension (3, 23, 24). In the present study, higher aortic PWV was found in the SHR rats when compared with the SHR.BN3 rats, and PWV was seen to further increase with age in the SHR strain, whereas no such progression was observed in SHR.BN3 animals (Fig. 2). Compared with SHR.BN3, SHR hearts, which likely experience greater aortic input impedance, demonstrate compensatory hypertrophic changes at 16 wk, as evidenced by increased fractional shortening, thickened LV walls, and a significantly greater RWT (Fig. 5). In response to further age-related increases in arterial stiffness, however, the SHR hearts undergo decompensatory changes at 32 wk, as indicated by significant decreases in fractional shortening and LV chamber dilatation with LV wall thinning and decreased RWT (Fig. 5, *A–G*). In contrast, the SHR.BN3 rats, which exhibit and maintain a lower aortic PWV, and thus likely lower aortic input impedance, preserve normal LV function and geometry with no significant change between 16 and 32 wk of age (Fig. 5).

It has been shown in a number of animal models and clinical studies that arterial stiffness precedes the development of hypertension (7, 25). Therefore, we hypothesized that the SHR.BN3 strain would not exhibit high blood pressure based on PWV and normal LV function and geometry at both 16 and 32 wk. Interestingly, despite not exhibiting any indicators of arterial stiffening as determined by PWV, the SHR.BN3 strain was found to have the same increase in BP with sustained hypertension that has been previously documented in the SHR model (26, 27). There were no differences in systolic, diastolic, or mean BP between the strains at any time point through 32 wk (Fig. 1). As such, BP is determined by both CO and peripheral vascular resistance. Physiologically, CO is a function of arterial tone in the elastic arteries, whereas peripheral vascular resistance is related to the arterial tone in muscular conduit arteries (20, 28). In the present study, we assessed PWV in the aorta, an elastic artery, and therefore, we would expect a tight relationship between PWV and CO in the SHR and SHR.BN3 rats. Given that mean BP is the same for both groups despite significantly lower PWV in the SHR.BN3 strain, we hypothesized, based on Darcy’s law, that CO output must be increased in the SHR.BN3 compared with SHR rats. To test this hypothesis, 16-wk-old rats underwent additional echocardiographic imaging for the determination of CO. Although heart rates were not different in these subsequent studies, SHR.BN3 rats demonstrated significantly greater stroke volume and thus significantly higher CO compared with SHR rats (Fig. 6). These data suggest that, in the face of reduced arterial stiffness, SHR.BN3 rats maintain an elevated BP through increased LV stroke work. These findings also suggest that the intrinsic autoregulatory mechanisms for maintaining BP at the predetermined set point in the SHR rats remain intact in the SHR.BN3 animals.

Normal arterial structure is also essential for appropriate function in the cardiovascular system. Thus, we hypothesized that the PWV differences could be due to genetic factors within the RNO3 QTL that may directly influence the structure of the arterial wall. The arterial wall is elastic and muscular with three distinct layers, each with a different composition and function (Fig. 3 and Fig. 4, *A* and *B*). The mechanical properties of the artery are largely dependent upon the composition of the tunica media and tunica adventitia, and there are many cell types within the adventitia. It is primarily, however, the fibroblasts that are responsible for the dramatic signaling cascades that result in the excess production or degradation of the matrix and proliferation of cells (6, 29–32). In healthy individuals, there is generally a higher ratio of elastin to collagen in the large conduit arteries that are considered arterially compliant with a normal elasticity index (33–35). The only significant structural difference noted between strains was total collagen and the presence of bundles in the SHR.BN3 strain (Fig. 4, *A–D*). Interestingly, regulation of the ECM environment is a homeostatic process that responds to injury, stress, or the development of CVD such as essential hypertension (7, 23, 36, 37). Disruption of the ECM environment as a result of essential hypertension results in the excess production of collagen, resulting in a vasculature that no longer exhibits the same elastic properties (33, 38, 39). A shift in the ratio of elastin to collagen results in an arterially stiff vasculature that disrupts blood flow, ultimately driving the ensuing vascular remodeling (9, 29, 34, 37–40). Disruptions to the structure of the supporting collagen network in the tunica adventitia often results in mechanical failure of the arterial wall (32). Although the SHR.BN3 vasculature appears to remodel as determined by the increase in collagen and the highly organized bundles, this seems to protect this strain from developing arterial stiffness. These results are unique compared with the development of arterial stiffness in other models of hypertension (8). These results suggest that the excess synthesis of collagen and the bundled organization in the SHR.BN3 strain likely strengthens the arterial tree and resists arterial distension with increasing blood pressure resisting an increase in media thickness (11, 22). As a result, this could increase CO, which can be further compounded by a variety of signaling methods including the enhancement of sympathetic tone (41) and changes in vasoconstriction and peripheral vascular resistance.

VSMCs are responsible for controlling the tone of the cardiovascular system by responding to stimuli that result in the dilation or constriction of the vasculature (20). These mechanisms control the diameter of the lumen, thus regulating pressure and flow within the cardiovascular system. VSMCs respond to a number of stimuli that include components of the ECM, environmental cues, and physiological events that produce sheer stress or stretching of the vasculature, which include stimuli like platelet-derived growth factor (PDGF), vascular endothelial growth factor (VEGF), integrins, nitric oxide, oxidative stress, angiotensin II, and others (20). The VSMCs can be synthetic or contractile in nature with phenotypic intermediates occurring most likely due to their distinct functions necessary for maintenance and/or physiological remodeling (42). Vasoconstriction occurs as a result of signaling cascades produced by intracellular Ca^2+^, endothelin, angiotensin II, or the activation of Rho-kinase (43). A diverse population of VSMCs is, therefore, able to respond to both the physiological and/or environmental stimuli in a complementary nature to maintain the overall arterial tone (42).

We hypothesized that the difference in arterial stiffness between the SHR and SHR.BN3 could be a result of VSMC phenotypic diversity found within the ECM of the artery controlling the vascular homeostasis (44). To address this question, we analyzed the percentage of proliferating VSMCs and the number of nuclei in the tunica media and tunica adventitia. Interestingly, there were no differences detected in proliferation or the total number of nuclei in adventitia or media of the SHR and SHR.BN3 (Fig. 4), indicating that the differences are not simply due to proliferation within the arterial wall. Evidence from Fig. 7 suggests that the differences in vascular tone associated with the RNO3 QTL could be due to the phenotypic plasticity of the VSMCs and the altered ECM organization and interactions (44). We found differences in ACTA2 in the VSMCs, suggesting that the contractile apparatus proteins may also play a direct role in the development of high blood pressure. Indeed, others have found that VSMC contractile abnormalities are enough to cause systemic hypertension including myosin phosphatase and RhoA/Rho kinase (45). However, the parameters controlling the epigenetic and gene expression landscape of the VSMCs remain to be determined.

In the present study, we have identified a series of physiological and anatomical differences associated with the RNO3 QTL region. Significant ECM differences were found in the arterial wall that affected structural and physiological measures associated with vascular tone, including collagen, PWV, LV geometry and function, and VSMC contractile apparatus proteins. As a result, our model provides a unique platform to further study the mechanisms controlling differences associated with vascular tone, as the current models of hypertension or vascular disorders all develop arterial stiffness before the progression of hypertension (8). Therefore, this model is essential for targeting the key genetic elements within the RNO3 QTL governing control of vascular remodeling, which result in the reorganization of the elastin and collagen components of the ECM and overall vascular tone. Insights into the mechanisms governing the arterial differences will aid in the development of new therapies to reduce and/or resolve the deleterious effects of arterial stiffening in cardiovascular disease risk and morbidity.

## GRANTS

This work was supported in part by the University of Toledo URAF Interdisciplinary Research Initiation Award (I-127366-01) to A.L.N.-K., M.P.M., and E.E.M.; the Alpha Omega Alpha Carolyn L. Kuckein Student Research Fellowship to N.G.H.; and an American Heart Association Postdoctoral Fellowship (09POST2400144) to E.E.M.

## DISCLOSURES

No conflicts of interest, financial or otherwise, are declared by the authors. 

## AUTHOR CONTRIBUTIONS

E.E.M. and A.L.N.-K. conceived and designed research; E.E.M., M.P.M., N.G.H., and A.L.N.-K. performed experiments; E.E.M., M.P.M., N.G.H., D.A.W., and A.L.N.-K. analyzed data; E.E.M., M.P.M., D.A.W., and A.L.N.-K. interpreted results of experiments; E.E.M., M.P.M., N.G.H., and A.L.N.-K. prepared figures; E.E.M. and A.L.N.-K. drafted manuscript; E.E.M., M.P.M., N.G.H., and A.L.N.-K. edited and revised manuscript; E.E.M., M.P.M., N.G.H., D.A.W., and A.L.N.-K. approved final version of manuscript. 
